# Diverse phenotypes in children with *PAX2*‐related disorder

**DOI:** 10.1002/mgg3.701

**Published:** 2019-05-06

**Authors:** Haiyue Deng, Yanqin Zhang, Huijie Xiao, Yong Yao, Xiaoyu Liu, Baige Su, Hongwen Zhang, Ke Xu, Suxia Wang, Fang Wang, Jie Ding

**Affiliations:** ^1^ Department of Pediatrics Peking University First Hospital Beijing China; ^2^ Department of Electron Microscopy Peking University First Hospital Beijing China

**Keywords:** children, new phenotypes, *PAX2* gene, renal coloboma syndrome

## Abstract

**Background:**

The aim of this study was to analyze the diverse phenotypes of children with *PAX2*‐related disorder so as to improve our understanding of this disease.

**Methods:**

The clinical data of ten children with *PAX2* mutations, detected by targeted region capture sequencing or whole‐exome sequencing, were retrospectively analyzed. Family members of index cases were verified by Sanger sequencing and family segregation analysis was performed.

**Results:**

The age of first symptom of 10 unrelated children (six girls and four boys) was 6.4 (ranged from postnatal day to 14.8) years old. Proteinuria, abnormal renal function, and structure were found in all patients. Renal hypoplasia and renal cysts were found in 10 of 10 and five of 10 cases, respectively. Three patients progressed to chronic kidney disease stage 5 and the onset age of end‐stage renal disease was 9.8–16.4 years old. *PAX2*‐related ocular abnormalities were found in five of seven cases and three patients were observed to have more than one ocular findings involved. In addition to diverse renal and ocular findings, new phenotypes including congenital ventricular septal defect, skeletal deformity (fourth metatarsal microsomia), ovarian teratoma, and relatively rare extrarenal manifestations such as growth retardation, gout, and microcephaly were also found. Three novel mutations were reported for the first time. *De novo* mutations occurred in all patients who were carried out segregation analysis. Patients with the same mutation had different manifestations. *PAX2*‐related disorder showed remarkable clinical variability and phenotypic heterogeneity.

**Conclusion:**

We firstly reported skeletal deformity (fourth metatarsal microsomia), ovarian teratoma, and congenital ventricular septal defect as new phenotypes of *PAX2*‐related disorder which enlarged the phenotypic spectrum. Gout was firstly reported as the onset symptom of *PAX2*‐related disorder. The diagnosis of *PAX2*‐related disorder should be considered without family history due to a much higher percentage of *De novo* mutations.

## 1 INTRODUCTION

*PAX2* (OMIM#167409) encodes a transcription factor that is expressed in the kidney, ureter, eye, ear, and central nervous system. It plays a key role in organ development and cellular regeneration (Harshman & Brophy, 2012). Renal coloboma syndrome (RCS, MIM**#**120330) with or without vesicoureteral reflux (VUR), also known as *PAX2*‐related disorder, is associated with *PAX2* mutation. *PAX2*‐related disorder is inherited in an autosomal dominant fashion and was originally characterized by renal hypoplasia or dysplasia and optic nerve abnormality. The rapid development of molecular testing has revealed a wide range of multisystem phenotypes associated with pathogenic *PAX2* variants and that hotspot mutations mostly occur in the paired domain encoded in exons 2–4 (Bower et al., 2012; Bower, Schimmenti, & Eccles, 1993). Ninety‐two percent of patients with *PAX2* mutation were reported to have abnormal renal structure or function, 77% to have ophthalmological abnormalities, and 7% to have hearing loss (Bower et al., 2012). Very few patients were reported to have central nervous system malformations (Schimmenti et al., 1997), developmental delay (Miyazawa et al., 2009), joint laxity (Iatropoulos et al., 2012), soft skin, or gout (Bower et al., 1993). The reported kidney and eye symptoms are diverse and nonspecific, and patients with the same mutation could present different phenotypes (Barua et al., 2014; Cheong et al., 2007; Nishimoto et al., 2001).

Here, we analyzed phenotypic manifestations in children with *PAX2* mutations. In addition to diverse renal and ocular findings, we report some new *PAX2*‐related disorder phenotypes which enlarged the phenotypic spectrum. Moreover, we highlight a remarkable level of clinical variability and phenotypic heterogeneity in children with *PAX2* mutations.

## 2 SUBJECTS AND METHODS

### 2.1 Ethical compliance

This study was performed in accordance with the Declaration of Helsinki and the Ethical Committee of Peking University First Hospital approved this project.

### 2.2 Patients

We enrolled 10 patients of *PAX2* (NM_003990.4) mutation from 423 individuals in our hereditary renal diseases registry database who were checked for genomic abnormality by targeted region capture sequencing (including 504 hereditary kidney diseases genes) or whole‐exome sequencing (WES) from August 2012 to May 2018. DNA sequences extracted from peripheral blood of family members were obtained via Sanger sequencing for segregation analysis. Patients with incomplete clinical data were excluded.

### 2.3 Research methods

We retrospectively reviewed clinical findings, including: the age and manifestation of the first symptoms; age of end‐stage renal disease (ESRD); kidney findings and extrarenal manifestations; and genetic testing and renal biopsy results. The glomerular filtration rate was estimated by correcting 24 hr creatinine clearance, divided into five stages: stage 1, >90 ml/(min·1.73 m^2^); stage 2, 60–89 ml/(min·1.73 m^2^); stage 3, 30–59 ml/(min·1.73 m^2^); stage 4, 15–29 ml/(min·1.73 m^2^); and stage 5, <15 ml/(min·1.73 m^2^) (Improving Global Outcomes CKD Work Group, 2013). Obesity was defined as a body mass index (BMI) greater than the 95th percentile of the Chinese children's reference value (Working Group on Obesity in China, 2005). Kidney size was judged by Loftus's report (Loftus, Gent, LeQuesne, & Metreweli, 1998). Hypoplastic kidneys were defined as those with ultrasound detected lengths less than the mean for the corresponding age minus two standard deviations. Out of 10 patients, the sequence data of nine were obtained through targeted region capture sequencing, and that of the remaining one was obtained using WES. For variants not yet reported, the pathogenicity was assessed as previously reported by our group (Wang et al., 2017).

## 3 RESULTS

### 3.1 Clinical characteristics

#### 3.1.1 General information

There were 10 patients enrolled in this study including six girls and four boys. The ages of the patients ranged from 2.2 to 15.4 years, and the average age was 10.8 years. We clearly knew the birthplace and ethnicity of nine of the 10 patients. These nine patients came from seven different provinces, one was Mongolian and the rest were of Han Chinese ethnicity.

The age of first symptom was 6.4 (range: postnatal day to 14.8) years old. Four cases (patients 1, 3, 7, and 8) first visited hospital because of abnormal renal function, four cases (patients 2, 5, 6, and 10) were identified following an abnormal urine test, one case (patient 4) had occasional renal cysts found during medical examination, and one case (patient 9) had articular pain.

Review of the fetal history revealed that only patient 5 had a recorded renal abnormality, with a unilateral kidney detected. Fetal descriptions of the remaining patients were unavailable.

#### 3.1.2 Kidney manifestations

Renal ultrasound results and kidney manifestations are shown in Table 1. All patients had abnormal kidney structure without ureterectasia. Renal hypoplasia was found in all cases, eight were bilateral and two were unilateral. Renal cysts were detected in five patients, two patients had multiple cysts and three had single cysts. Proteinuria and renal dysfunction were also found in all patients. Among them, nephrotic‐level proteinuria (≥50 mg/kg/24 hr) was identified in six cases. Three patients progressed to chronic kidney disease (CKD) stage 5, and the onset age of ESRD occurred at 9.8–16.4 years old. For patients 3 and 8, it took 3.4–6.4 years to progress from CKD stage 3 to CKD stage 5.

**Table 1:** Clinical features and *PAX2* mutations observed in 10 pediatric patients

Patient (No.)	1	2	3	4	5	6	7	8	9	10
Gender	F	M	F	F	M	M	M	F	F	F
Onset age(years)	9.8	4.3	7.8	4	5	9.7	2.1	10	14.8	postnatal day
*Renal manifestations*										
Age of abnormal Scr/Onset of ESRD (years)	9.8/9.8	4.3/‐	7.8/11.2	10.9/‐	10.2/‐	11/‐	2.1/‐	10/16.4	15.2/‐	postnatal day/‐
Urinary findings	PU, GU	PU, mHU	PU	PU	PU, mHU	PU	PU, HCU	PU	PU	PU, mHU
24h urinary protein (g/24hr)	3.96	0.25	1.34	2.24	2.72	0.43	1.92	2.14	2.42	0.99
Nephrotic‐level proteinuria	+	‐	‐	+	+	‐	+	+	‐	+
GFR (ml/min/1.73 m^2^)	2.9	64	15	41.7	59	59.6	29.4	5.2	25.1	68
*Renal gross morphology*										
Age at renal ultrasound (years)	10.1	5.7	11.2	13.1	10.3	11.8	2.1	16.4	15.2	2.2
Bilateral renal hypoplasia	+	+	+	‐	+	+	+	+	+	‐
Unilateral renal hypoplasia	‐	‐	‐	+(right)	‐	‐	‐	‐	‐	+(right)
Multiple renal cysts	‐	‐	‐	+(right)	‐	‐	+(bilateral)	‐	‐	‐
Single renal cysts	‐	‐	‐	‐	‐	+(left)	‐	+(right)	‐	+(right)
*Renal biopsy*										
Age at renal biopsy(years)	NA	5.7	NA	NA	10.3	11.8	NA	NA	15.2	NA
BMI (95 percentile)	NA	16.4 (17.9)	NA	NA	17.4 (22.2)	20.9 (23.5)	NA	NA	26.4 (24.8)	NA
Pathology	NA	Oligomeganephronia combined with atypical MN	NA	NA	MsPGN combined with glomerular hypertrophy and chronic renal tubulointerstitial nephropathy	Focal proliferative sclerosing purpura nephritis with glomerular hypertrophy	NA	NA	FSGS with chronic tubulointerstitial nephropathy	NA
*Ophthalmological findings*										
Morning glory anomaly	‐	NA	‐	+	‐	NA	NA	‐	+	‐
Abnormal retinal pigment epithelium	‐	NA	‐	‐	+	NA	NA	‐	+	‐
Macular coloboma	‐	NA	‐	+	‐	NA	NA	‐	‐	‐
Microphthalmia	‐	NA	‐	‐	‐	NA	NA	+	‐	‐
Amblyopia	‐	NA	‐	‐	+	NA	NA	‐	‐	‐
Strabismus	‐	NA	+	+	‐	NA	NA	‐	‐	‐
*Other findings*										
Microcephaly	‐	‐	‐	+	‐	‐	‐	‐	‐	‐
Developmental delay	‐	‐	‐	+	‐	‐	‐	‐	‐	‐
Growth retardation	‐	‐	‐	‐	‐	‐	+	‐	‐	‐
Metatarsal microsomia	‐	‐	‐	‐	‐	+	‐	‐	‐	‐
Congenital ventricular septal defect	‐	‐	‐	‐	‐	‐	‐	+	‐	‐
Ovarian teratoma	‐	‐	‐	+	‐	‐	‐	‐	‐	‐
Gout	‐	‐	‐	‐	‐	‐	‐	‐	+	‐
*PAX2* * mutation*										
Nucleotide alteration	c.88G > T	c.76dupG	c.76dupG	c.76delG	c.76delG	c.272C > T	c.343C > T	c.418C > T	c.418C > T	c.410 + 1G＞A
Deduced protein change	p. Gly30Cys	p. Val26Glyfs*28	p. Val26Glyfs*28	p. Val26Cysfs*3	p. Val26Cysfs*3	p. Ala91Val	p. Arg115*	p. Arg140Trp	p. Arg140Trp	‐
Location	EX2	EX2	EX2	EX2	EX2	EX3	EX3	EX4	EX4	IVS3
Zygosity (Segregation)	Het (NA)	Het (NA)	Het (N)	Het (N)	Het (N)	Het (NA)	Het (NA)	Het (N)	Het (N)	Het(N)
Reference	This study	Sanyanusin P (Sanyanusin, McNoe, Sullivan, Weaver, & Eccles, 1995)	Sanyanusin P (Sanyanusin et al., 1995)	Heidet L (Heidet et al., 2017)	Heidet L (Heidet et al., 2017)	This study	Schimmenti LA (Schimmenti, Manligas, & Sieving, 2003)	Negrisolo S.^a^	Negrisolo S.^a^	This study

Note:

F, female; M, male; PU, proteinuria; mHU, microscopic hematuria; HCU, hypercalciuria; GU, glycosuria; GFR: glomerular filtration rate, use 24h creatinine clearance to evaluate; MN, membranous nephropathy; MsPGN: mesangial proliferative glomerulonephritis; FSGS: focal segmental glomerulosclerosis; BMI: body mass index; NA: not available; *N*: Mutations could not be found in father or mother. Accession no: NM_ 003990.4.

a Reference Leiden Open Variation Database (LOVD).

Renal biopsies were performed in four patients, with varying results (Table 1). Most biopsies were analyzed using light and electron microscopy, except for patient 9 for whose electron microscopy results were not available. Focal segmental glomerular sclerosis (FSGS) was only observed in patient 9. Glomerular hypertrophy was observed in two patients. Obesity‐related nephropathy could be excluded for these two patients as their calculated BMIs did not indicate obesity. Considering the performance of purpura, the main pathological diagnosis for patient 6 was focal proliferative sclerosing purpuric nephritis.

#### 3.1.3 Extrarenal manifestations

Seven cases finished ophthalmic examinations and the ocular manifestations are summarized in Table 1. Patient 1 was diagnosed as having binocular retinopathy due to hypertension and patient 10 had normal results. Five of seven children had *PAX2*‐related ophthalmological abnormalities. Morning glory optic discs have been reported as a classic RCS ocular change (Bower et al., 2012) and two of our patients had this anomaly. Additionally, there were extensive involvements outside the optic discs, including abnormal retinal pigment epithelium and macular coloboma. Meanwhile, microphthalmia, amblyopia, and strabismus were also observed. Three patients were reported to have more than one ocular finding.

**Figure 1:**
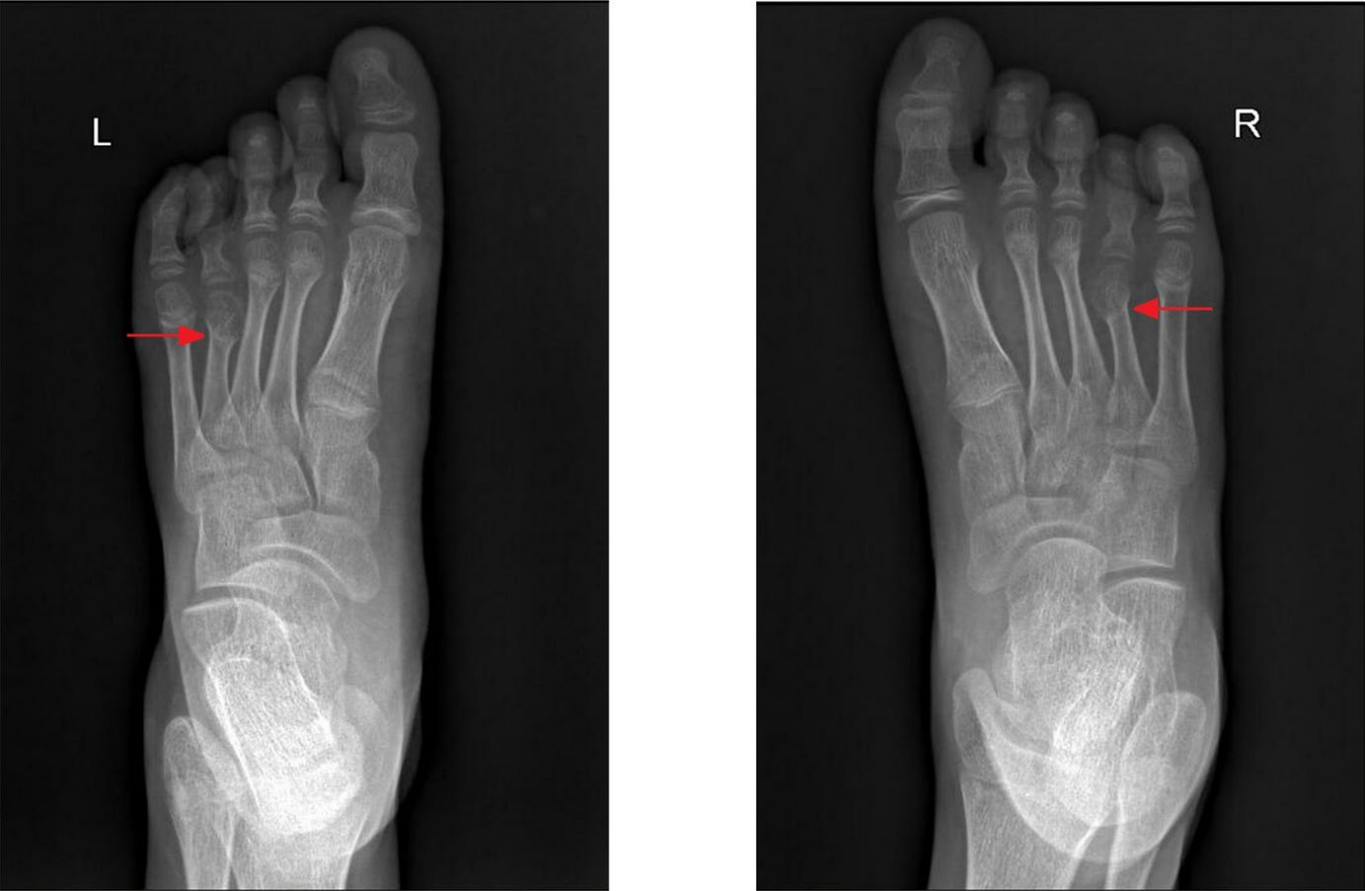
The Biped X‐ray frontal film results of patient 6. The red arrow indicates fourth metatarsal microsomia. L: Left; R: Right

**Figure 2:**
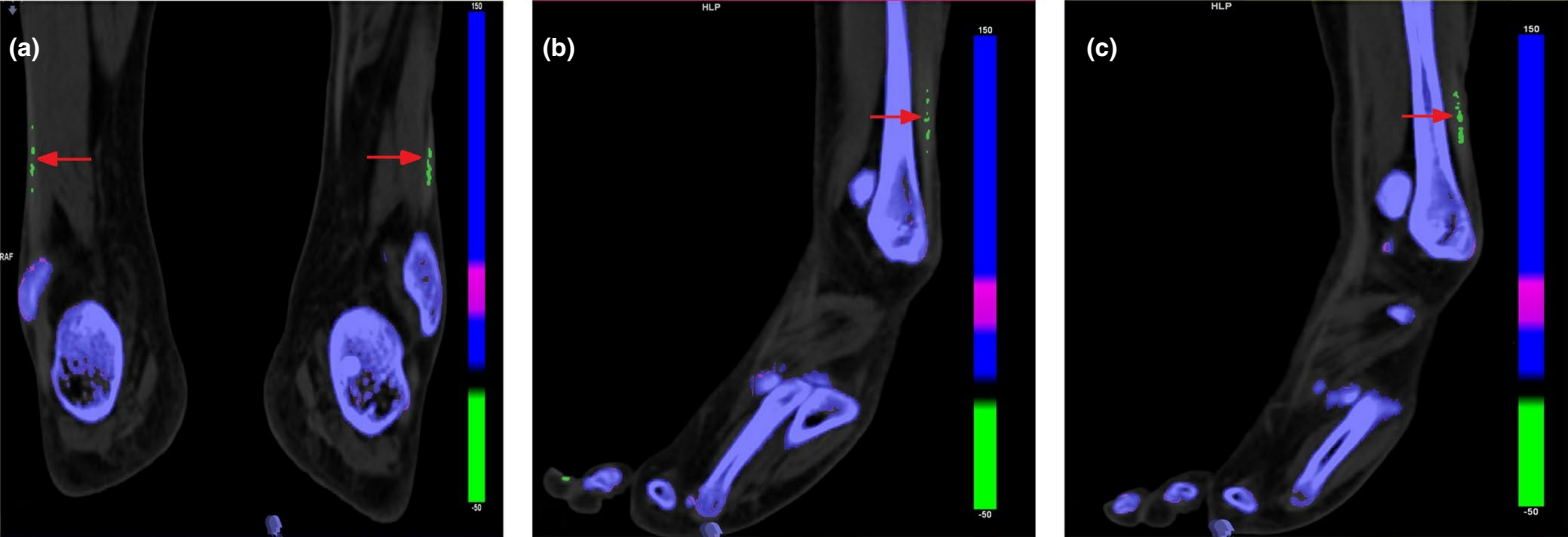
The Biped dual‐energy CT post‐processing results for patient 9. The red arrow indicates gout nodules. a: Bilateral; b: Right; c: Left

Some patients also had relatively rare extrarenal manifestations. Patient 4 was diagnosed with microcephaly and ovarian teratoma. She also had developmental delay since infancy, and the Wechsler intelligence scale for children suggested a mental deficiency. This child was born by cesarean section due to abnormal fetal heart rate and amniotic fluid contamination. Her Apgar score was seven to eight. Patient 6 had fourth metatarsal microsomia of bipedal with normal bone mineral density (Figure 1). Patient 7 had growth retardation. Patient 8 had a congenital ventricular septal defect. The first symptom of patient 9 was joint swelling and pain with elevated serum creatinine and uric acid. Biped dual‐energy computed tomography post‐processing imaging showed that uric acid was deposited in the lower part of the fibula, and gout nodules were diagnosed (Figure 2).

### 3.2 *PAX2* mutations

**Figure 3:**
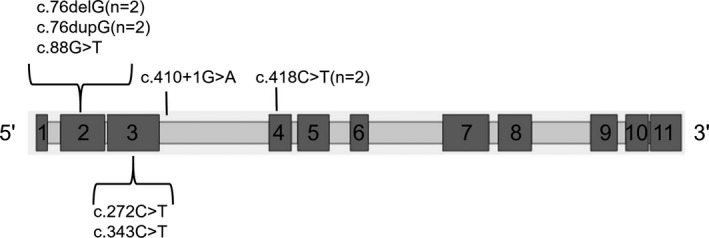
Distribution of *PAX2* gene mutations in 10 patients. The boxes represent exons (to scale)

Seven different mutations, most of which occurred in exons 2–4, were found in the 10 patients (Table 1; Figure 3). Mutations c.88G > T, c.272C > T, and c.410 + 1G>A were novel and not previously reported in the Human Gene Mutation Database (HGMD, http://www.hgmd.cf.ac.uk/ac/search.php) nor in ClinVar (https://www.ncbi.nlm.nih.gov/clinvar/). Bio‐infomercial analysis is shown in Table 2. The c.76dupG frame shift mutation and the c.343C > T nonsense mutation have been identified as *PAX2* mutational hotspots and have been reported 57 and six times, respectively, in the Leiden Open Variation Database (http://www.lovd.nl/3.0/home). Of 10 patients, segregation analysis was performed for six of the 10 patients using Sanger sequencing. Mutations could not be detected in their first‐degree relatives, indicating that these mutations may have occurred *de novo* mutation.

**Table 2:** Bio‐infomercial analyses of *PAX2* mutations

Nucleotide Change	Predicted Effect on Protein	Database		Frequency in normal control		In silico analysis					
						SIFT		Mutation Taster		Polyphen2	
		ClinVar	HGMD	1000G	ExAC	Prediction	Score	Prediction	Score	Prediction	Score
c.88G > T	p. Gly30Cys	–	–	–	–	deleterious	0.00	disease causing	0.99	Probably damaging	1.00
c.76dupG	p. Val26Glyfs*28	Pathogenic	Disease‐causing mutation	–	–	–	–	disease causing	1.00	–	–
c.76delG	p. Val26Cysfs*3	Pathogenic	Disease‐causing mutation	–	–	–	–	disease causing	1.00	–	‐
c.272C > T	p. Ala91Val	–	–	–	–	deleterious	0.01	disease causing	0.99	Probably damaging	1.00
c.343C > T	p. Arg115*	–	Disease‐causing mutation	–	–	–	–	disease causing	1.00	–	–
c.418C > T	p. Arg140Trp	–	Disease‐causing mutation	–	–	deleterious	0.00	disease causing	0.99	Probably damaging	1.00
c.410 + 1G>A	–	–	–	–	–	–	–	disease causing	1.00	–	–

Note

Accession no: NM_ 003990.4

### 3.3 Phenotypic heterogeneity

Most *PAX2* mutations detected in our patients occurred in the paired domain, known for its DNA‐binding properties. All patients had progressive renal dysfunction and renal hypoplasia without ureterectasia. However, it was difficult to identify the correlation between the expressed phenotypes and the specific location of the mutation. Patients 2 and 3 had the same mutation, as did patients 4, 5 and patients 8, 9. However, their phenotypes were inconsistent. The onset age for patients 2 and 3 was 4.3 and 7.8 years, respectively, and the kidney progression showed some diversity. Patient 2 was in CKD stage 2 with a disease course of 1.4 years, and renal pathology confirmed oligomeganephronia combined with atypical membranous nephropathy (MN), while patient 3 was in CKD stage 5 with a 3.4‐year‐disease course, with bilateral renal hypoplasia, and ocular strabismus. The renal gross morphology in patients 4 and 5 differed. Right renal hypoplasia and multiple cysts in patient 4 while bilateral renal hypoplasia in patient 5. Patient 4 also combined with some relatively rare manifestations such as microcephaly, developmental delay, and ovarian teratoma. The onset symptoms for patients 8 and 9 completely differed and were abnormal renal function and articular pain, respectively. The ocular manifestations were also diverse, from microphthalmia to abnormal retinal epithelium pigment. They also had nonrenal and nonocular findings, such as congenital ventricular septal defect in patient 8, and gout and hyperuricemia in patient 9. Therefore, the phenotypic manifestations of the same mutation were heterogeneous.

## 4 DISCUSSION

Here, we reported diverse renal and extrarenal phenotypes associated with *PAX2* mutations. All patients had proteinuria with abnormal kidney structure and function. However, only two patients presented with classic signs of RCS: renal disorder accompanied with morning glory optic discs. Most mutations were located in exons 2–4.

First, we reported some rare extrarenal phenotypes involved multiple systems in our patients including skeletal deformity (fourth metatarsal microsomia), ovarian teratoma, and congenital ventricular septal defect. *PAX2* may be involved in the development of the skeleton and ovary, and in heart morphogenesis (Prathibha & Senthilkumaran, 2016; Röttinger et al., 2008; Wang, Lan, Cho, Maltby, & Jiang, 2005). Only one child had previously been reported with a *PAX2* mutation and a skeletal deformity (congenital camptodactyly) (Liu, Wang, Huang, & Yu, 2018). We believed these skeletal, ovarian, and heart defects may be new *PAX2*‐related disorder phenotypes and we enlarged the phenotypic spectrum. Abnormal central nervous system phenotypes (microcephaly and developmental delay), growth retardation, and gout have been reported previously (Cunliffe et al., 1998; Megaw, Lampe, Dhillon, Yoshida, & Wright, 2013; Schimmenti et al., 1997). These relatively rare phenotypes were also found in our study, whereas gout was firstly reported as the onset symptom of *PAX2*‐related disorder, and clinicians should consider *PAX2*‐related disorder in children with gout accompanied with unexplained impaired kidney function.

Renal hypoplasia was detected in all patients, and renal cysts was detected in 5 out of 10 patients in our study, which is higher than 65% and 8%, respectively, reported previously (Bower et al., 2012). It was also reported that VUR was the second most common renal finding (14%) associated with *PAX2* mutation (Bower et al., 2012). However, a Korean study indicated that six renal coloboma syndrome children showed no evidence of VUR (Cheong et al., 2007). None of the patients included in our study finished retrograde ureteropyelography, so their VUR status was unknown. However, ultrasound results suggested no ureterectasia. Compared to the frequencies reported for Europeans and Americans, renal hypoplasia and renal cysts may be more common in China and Asians may have a lower rate of VUR.

In our report, the onset age of CKD Stage 5 was between 9.8–16.4 years. In a previous study, the onset of CKD Stage 5 was reported to occur up to the age of 79 years (Bower et al., 2012). The progression to ESRD differed, patient 3 in our study, progressed to ESRD at 11.2 years old with c.76dupG (p.Val26Glyfs*28), while a patient with the same mutation, reported by another group, had not progressed to ESRD at 52 years old (Iwafuchi et al., 2016). These results indicate that kidney dysfunction progressed variously even for patients with the same mutation.

There was only one patient in our cohort who had FSGS. *PAX2* may lead to FSGS through dysregulating WT1, a nuclear protein expressed in podocytes (Gebeshuber et al., 2013; Lipska et al., 2013). Four percent of patients with adult‐onset FSGS harbor a *PAX2* mutation (Barua et al., 2014). Moreover, 60% of adult patients with *PAX2* mutations have FSGS (Okumura et al., 2015). The remaining findings (oligomeganephronia combined with atypical membranous nephropathy, and mesangial proliferative glomerulonephritis) may be due to the younger age at renal biopsy or the diversity of pathological results with *PAX2* mutation, some of which have been reported (Okumura et al., 2015; Salomon et al., 2001). In addition, patient 6 was diagnosed with focal proliferative sclerosing purpura nephritis. While it has been noted that in most purpura nephritis patients, more advanced morphological lesions are reflected by a more marked proteinuria (Halling, Söderberg, & Berg, 2005), patient 6 only had a 1.5‐year disease course of mild proteinuria. A simple history of purpura is insufficient to explain the renal pathology in this patient. A potential combination of *PAX2*‐related disorder may account for this phenotype.

The morning glory optic disc is a typical ocular manifestation associated with RCS (Schimmenti, 2011). More than half of our patients (5 of 7 cases) had eye involvement and only two patients had typical change. Ophthalmological findings varied from strabismus to microphthalmia to macular coloboma. The range and severity of eye lesions also varied greatly. This was consistent with previous reports (Bower et al., 2012; Cheong et al., 2007) and highlights the need to pay attention to atypical ocular changes when diagnosing the disease.

Although most identified mutations were located in the paired‐domain, we identified tremendous variability in renal and ocular phenotypes. The phenotypic manifestations of the mutations were heterogeneous, with the same mutation showing different symptoms. For example, two of our cases possessed heterozygous c.76dupG mutation (p.Val26Glyfs*28) in exon 2, that had been reported previously (Iwafuchi et al., 2016) and was responsible for the animal model of human RCS (Porteous et al., 2000). Compared to patients previously reported, renal pathology in our patients ranged from oligomeganephronia to FSGS, with or without optic coloboma. Another group also reported discordant phenotypes in monozygotic twins with the same mutation (Iatropoulos et al., 2012). It has been proposed that haploinsufficiency and epigenetic factors may play critical roles in the expression of clinical features (Benetti et al., 2007; Iatropoulos et al., 2012). The origin of the mutations in our cohort was confirmed via parental Sanger sequencing in six of 10 cases and all mutations were deemed *de novo*. The ratio of *de novo* mutations identified here is higher than previously reported (Bower et al., 2012). However, due to the lack of sibling data in this analysis, we were unable to determine whether parental germline mosaicism was involved. This greatly complicated prenatal diagnosis and genetic counseling procedures.

Our study had several limitations. The kidney structures during the fetal period had not been clearly recorded for most patients, and it was impossible to determine whether the patients had renal dysplasia during this period. The results of pure tone audiometry and retrograde ureteropyelography were not available for most patients, and whether patients had hearing loss and VUR was unknown.

In summary, we firstly reported skeletal deformity (fourth metatarsal microsomia), ovarian teratoma, and congenital ventricular septal defect as new phenotypes of *PAX2*‐related disorder which enlarged the phenotypic spectrum. Gout was firstly reported as the onset symptom of *PAX2*‐related disorder. The diagnosis of *PAX2*‐related disorder should be considered without family history due to a much higher percentage of *de novo* mutations.

## CONFLICT OF INTEREST

The authors declare that they have no conflict of interest.
